# Infection prevention and control of highly infectious pathogens in resource-limited countries: an experience from Marburg viral disease outbreak in Kagera Region - Tanzania

**DOI:** 10.1186/s12879-024-09508-5

**Published:** 2024-06-24

**Authors:** Erick Kinyenje, Joseph Hokororo, Ruth Ngowi, Michael Kiremeji, Elice Mnunga, Angela Samwel, Erasto Sylvanus, Emmanuel Mnken, Missana Yango, Mikidadi Mtalika, Vida Mmbaga, Noel Saitoti, Alex Malecha, Faith Kundy, Martin Rwabilimbo, Issessanda Kaniki, Godwin Mwisomba, Erica Charles, Patrick Mughanga, Mary Kitambi, Radenta Paul, Erick Richard, Atuganile Musyani, Irene Rabiel, Gift Haule, Laura Marandu, Emmanuel Mwakapasa, Gerald Manasseh, Calvin Sindato, Medard Beyanga, Eliakimu Kapyolo, Frank Jacob, Jonathan Mcharo, Mary Mayige, Faraja Msemwa, Grace Saguti, George Kauki, Janeth Masuma, George Mrema, Mugendi Kohi, Zabulon Yoti, Michael Habtu, William Mwengee, Kokuhabwa Mukurasi, Wangeci Gatei, Paschal Ruggajo, Elias Kwesi, Eliudi Eliakimu, Pius Horumpende, Grace Magembe, Tumaini Nagu

**Affiliations:** 1 Health Quality Assurance Unit, Ministry of Health, P. O. Box 743, Dodoma, Tanzania; 2 Emergency Preparedness and Response Unit, Ministry of Health, P. O. Box 743, Dodoma, Tanzania; 3 Department of Pediatrics and Child Health, Bugando Medical Center, P. O. Box 1370, Mwanza, Tanzania; 4 Health Promotion Section, Department of Preventive Services, Ministry of Health, P. O. Box 743, Dodoma, Tanzania; 5 Department of Internal Medicine, Dodoma Regional Referral Hospital, P. O. Box 904, Dodoma, Tanzania; 6 Wildlife Research Institute, Kingupira Wildlife Research Centre, P. O. Box 16, Utete- Rufiji, Tanzania; 7 Epidemiology Section, Department of Preventive Services, Ministry of Health, P. O. Box 743, Dodoma, Tanzania; 8 Department of Internal Medicine, Iringa Region Referral Hospital, P. O. Box 1260, Iringa, Tanzania; 9 Bukoba Regional Referral Hospital, P. O. Box 299, Bukoba, Kagera Tanzania; 10 Regional Secretariat, P.O Box 256, Kagera Bukoba, Tanzania; 11 Mirembe National Mental Health Hospital, P. O. Box 910, Dodoma, Tanzania; 12 Singida Regional Referral Hospital, P. O. Box 104, Singida, Tanzania; 13 Department Emergency Medicine, Bugando Medical Center, P. O. Box 1370, Mwanza, Tanzania; 14 Amref Health Africa in Tanzania, P. O. Box 2773, Dar es Salaam, Tanzania; 15 Quality Assurance Department, Bugando Medical Center, P. O. Box 1370, Mwanza, Tanzania; 16 President’s Office - Regional Administration and Local Government, Dodoma, Tanzania; 17 Department of Clinical Research, National Institute for Medical Research, Tabora Medical Research Centre, P. O. Box 482, Tabora, Tanzania; 18 National Public Health Laboratory, P. O. Box 60000, Dar es Salaam, Tanzania; 19 Department of Clinical Research, Dodoma Medical Research Centre, National Institute for Medical Research, P. O. Box 805, Dodoma, Tanzania; 20 National Institute of Medical Research, Head Quarters, P. O. Box 2769, Dar es Salaam, Tanzania; 21 World Health Organization, Dar es Salaam, Tanzania; 22 Centers for Disease Control and Prevention, Dar es Salaam, Tanzania; 23 Directorate of Curative Services, Ministry of Health, P. O. Box 743, Dodoma, Tanzania; 24 Unit of Research Coordination, Directorate of Curative Services, Ministry of Health, P. O Box 743, Dodoma, Tanzania; 25 Department of Research and Innovation, Peoples’ Defence Forces (TPDF) es Salaam, Lugalo Military College of Medical Sciences (MCMS) and General Military Hospital (GMH), P. O. Box Dar, Dar es Salaam, Tanzania; 26 Bukoba District Council Hospital, Kagera Region, P. O. Box 491, Bukoba, Tanzania; 27 Chato Zonal Hospital, P. O. Box 43, Chato- Geita, Tanzania; 28 University of Iringa, P. O. Box 200, Iringa, Tanzania; 29 World Health Organization Regional Office for Africa, Brazzaville, Congo; 30 Office of Permanent Secretary, Ministry of Health, P. O. Box 743, Dodoma, Tanzania; 31 Office of Chief Medical Officer, Ministry of Health, P. O. Box 743, Dodoma, Tanzania

**Keywords:** Marburg viral disease, Viral hemorrhagic fever, Infection prevention and control, Kagera, Tanzania

## Abstract

Marburg viral disease (MVD) is a highly infectious disease with a case fatality rate of up to 90%, particularly impacting resource-limited countries where implementing Infection Prevention and Control (IPC) measures is challenging. This paper shares the experience of how Tanzania has improved its capacity to prevent and control highly infectious diseases, and how this capacity was utilized during the outbreak of the MVD disease that occurred for the first time in the country in 2023.

In 2016 and the subsequent years, Tanzania conducted self and external assessments that revealed limited IPC capacity in responding to highly infectious diseases. To address these gaps, initiatives were undertaken, including the enhancement of IPC readiness through the development and dissemination of guidelines, assessments of healthcare facilities, supportive supervision and mentorship, procurement of supplies, and the renovation or construction of environments to bolster IPC implementation.

The official confirmation and declaration of MVD on March 21, 2023, came after five patients had already died of the disease. MVD primarily spreads through contact and presents with severe symptoms, which make patient care and prevention challenging, especially in resource-limited settings. However, with the use of a trained workforce; IPC rapid needs assessment was conducted, identifying specific gaps. Based on the results; mentorship programs were carried out, specific policies and guidelines were developed, security measures were enhanced, all burial activities in the area were supervised, and both patients and staff were monitored across all facilities. By the end of the outbreak response on June 1, 2023, a total of 212 contacts had been identified, with the addition of only three deaths. Invasive procedures like dialysis and Manual Vacuum Aspiration prevented some deaths in infected patients, procedures previously discouraged.

In summary, this experience underscores the critical importance of strict adherence to IPC practices in controlling highly infectious diseases. Recommendations for low-income countries include motivating healthcare providers and improving working conditions to enhance commitment in challenging environments. This report offers valuable insights and practical interventions for preparing for and addressing highly infectious disease outbreaks through implementation of IPC measures.

## Introduction

Marburg viral disease (MVD), formerly known as Viral Hemorrhagic Fever (VHF), is a very fatal disease with an approximate case fatality rate up to 90% [1–3]. The disease is caused by the Marburg virus, a member of the Filoviridae family, with transmission occurring through contact with the *Egyptian rousette* fruit bat [4–6]. MVD is acquired through direct contact with infected body fluids (such as through broken skin or mucous membranes in the eyes, nose, or mouth). The effect of MVD is more pronounced in resource-limited countries for various reasons, one of which is the limited capacity to implement Infection Prevention and Control (IPC) measures. For example, while the first case of MVD was detected in Europe in 1967 [7]; the majority of deaths and cases occurred in low-income countries subsequently [1, 8, 9].

The MVD, like other VHFs, often presents with severe watery diarrhoea, vomiting, and bleeding. This bleeding often occurs from various places such as the nose, gums, and vagina. These symptoms make it challenging to provide proper care to the patient as well as to prevent and control the disease within the community and among healthcare workers. For multiple reasons including these challenges, medical guidelines advise against performing invasive procedures [10] such as dialysis to patients who developed acute renal failure in MTUs or a pregnant woman who lost a pregnancy and requires Manual Vacuum Aspiration (MVA). The pressing concern lies in how these two patients would survive in a resource-limited country, such as Tanzania, particularly when there is a scarcity of viable alternative procedures within MVD Treatment Units (MTUs). Strict adherence to IPC interventions is necessary to achieve treatment goals. In our case, we had one patient requiring dialysis due to acute renal failure and another patient requiring MVA after an incomplete abortion.

World Health Organization (WHO) and other health partners developed a multisectoral process known as Joint External Evaluation (JEE) to assess countries’ capacities to prevent, detect, and rapidly respond to public health risks including emerging and re-emerging diseases such as MVD [11]. IPC is one of the five capacities required for responding to public health risks outlined in the third version of JEE [12], others are health emergency management, linking public health and security authorities, health services provision (resilient national health systems), and risk communication and community engagement. Tanzania was among the first countries to participate in the JEE in 2016 for the very first time. During this evaluation, it became apparent that the country had insufficient capacity to enforce IPC measures [13–15]. Tanzania, being a member state of the WHO, has been providing State Party Self-Assessment Annual Reports (SPARs) in addition to the JEE. These reports showcase the level of progress made in implementing the International Health Regulations IHR (2005) across 13 capacities required to detect, assess, notify, report, and respond to public health risks. One of these capacities is IPC. In 2017, the initial report was published and identified notable deficiencies in IPC, which were also highlighted in the JEE [16]. Consequently, Tanzania had to implement numerous measures to prepare the country to detect, assess, report and respond to public health risks based on various findings, including those mentioned above.

This study describes the key measures undertaken by the country to enhance the IPC situation within six years following the JEE. It outlines the successful outcomes attained and highlights their instrumental role in swiftly containing the outbreak of MVD that was declared in March 2023 in Kagera Region in Tanzania.

### Advancing in-country infection prevention and control capacity: a six-year progress (2018–2023)

The management of IPC measures in healthcare facilities in Tanzania began a long time ago (including strengthening implementation of IPC using the “*standard-based management and recognition*” approach [17]); however, there has been an increased focus during the six years that followed 2017. After several internal and external assessments, including the JEE, the Ministry undertook a step to prepare a multisectoral National Action Plan for Health Security [13] to address identified shortcomings. The plan in the IPC area included the revision of existing guidelines in 2018 and the formulation of new standard operating procedures (SOPs) in 2020. These updates incorporated new elements on addressing the prevention and control of emerging and re-emerging diseases. More specifically, the National IPC Guidelines for Health Care Services and SOPs detailed recommended practices for cleaning equipment used in healthcare (decontamination of instruments), safety precautions during high-risk diseases like Ebola, and VHF, as well as strategies to prevent Healthcare-Associated Infections (HAIs), such as Surgical Site Infections (SSIs) [18].

Improvement in guidelines and SOPs provided an opportunity at the national level to reach and train as many healthcare workers as possible. Out of the nearly one hundred thousand healthcare workforce available in the country [19], 4,947 received training. In the affected region of Kagera, over 540 workforce had been trained among approximately 3,000 available [19]. Trained individuals aimed to cascade training to colleagues who had not been covered, and the respective regions utilized developed guidelines and SOPs for regional training and mentorship.

Another key task was to ensure guidelines were accessible to all facilities. Therefore, training was accompanied by the dissemination of IPC guidelines to all healthcare facilities using a multifaceted approach [20] to facilitate healthcare workers’ (HCWs) compliance with IPC measures [21]. Following dissemination, facilities were assessed to check whether they were adhering to the guidelines. Selected referral hospitals were targeted first, followed by primary healthcare facilities based on resource availability and the risk of receiving suspected cases of infectious diseases. Prior to the outbreak of MVD, Bukoba RRH (the highest referral point for all healthcare facilities in the affected region) underwent two rounds of assessments. In general, the results of assessments revealed that each facility had made progress, with overall compliance rates increasing from 46% in 2021 to 64% in 2022 in adhering to IPC measures [22]. Furthermore, the Ministry provided guidelines for hospitals to conduct internal assessments at least quarterly.

Subsequently, the assessment revealed weaknesses in the management of Quality Improvement (QI) initiatives among service providers. Consequently, the Ministry decided to enhance the capacity of these personnel through training by utilizing existing guidelines for QI [23]. Apart from IPC, it was also essential to establish management structures that support IPC. The guidelines directed the establishment and strict management of QI structures from the national level down to the facility level [24–27]. Quality Improvement Teams (QITs) had the responsibility of coordinating and overseeing the performance of other quality-related functions at the facility, including the IPC committee. At the national level, their role includes the conduct of assessments at various levels, including facilities.

Bukoba RRH, where MVD patients were treated, had become an excellent example of effective QIT performance with an established functional IPC committee. For example, two years before MVD outbreak, the hospital started to conduct series of internal assessments to identify deficiencies in IPC capacities. One of the key gaps identified was a high prevalence of SSIs. The December 2021 and January 2022 assessments found that 15 out of 166 surgery patients at the hospital acquired SSIs. By using the QI approach of the “5 WHYs,” [23] the root causes were discovered and resolved. After six months of implementing the set measures, SSI rates dropped to 5%; equivalent to 35 SSIs out of a total of 715 surgical clients. All these efforts were aimed at improving IPC measures for HAIs, antimicrobial resistance (AMR) [28, 29] as well as emerging and reemerging infections including outbreaks like MVD.

Extending further initiatives, in the year 2021, the Ministry of Health decided to establish a Monitoring and Evaluation (M&E) framework for IPC indicators, aiming to ensure the continuous management of the performance of selected IPC indicators. These indicators are based on different key IPC components that are critical elements in controlling emerging and re-emerging infectious diseases. These components include the functionality of QI and IPC teams in the facilities; cascading training to HCWs; surveillance of healthcare-associated infections such as surgical infections; waste management; water, sanitation and hygiene (WASH) services (including water availability in healthcare facilities); implementation of handwashing; and decontamination. From November 2021 to August 2023, a total of 695 HCWs from 114 facilities were trained on the IPC M&E system. The goal was for trained HCWs to teach others. These HCWs came from all referral hospitals, constituting 42 out of 42 (100%) of the target, and from 37 council hospitals, representing 20.8% of the target of 178 council hospitals. However, due to budget constraints, HCWs from private hospitals that are not designated as referral points were not trained [30]. Out of the trained facilities (114), 96(84%) reported implementing IPC interventions through the District Health Information System (DHIS-2) at the start of the outbreak.

### Declaration of the outbreak of Marburg in Kagera, Tanzania

In a recent turn of events, Tanzania faced a severe test of its preparedness for infectious disease outbreaks when an MVD outbreak struck the nation on March 16, 2023. This outbreak, which was officially declared on March 21, 2023, was the first in the country [31], and Tanzania became the fifth African nation to declare infections of this disease. MVD, first identified in Germany in 1967, has afflicted ten countries worldwide, claiming the lives of 80% of those infected [1, 3].

The declaration by Hon. Minister for Health Ms Ummy A. Mwalimu (Member of Parliament-MP) came after an intensive five-day investigation of an “unknown deadly infectious disease” that had already claimed the lives of five individuals. At the conclusion of the outbreak, the total number of cases was nine, with six of them resulting to fatalities. Laboratory testing confirmed all cases except for the initial index case. The index case, a young fisherman from Goziba Island in Lake Victoria, is suspected to have infected four relatives, three of whom were among the fatalities. Additionally, two healthcare workers were among the affected individuals, resulting in one fatality.

**Fig. 1:**
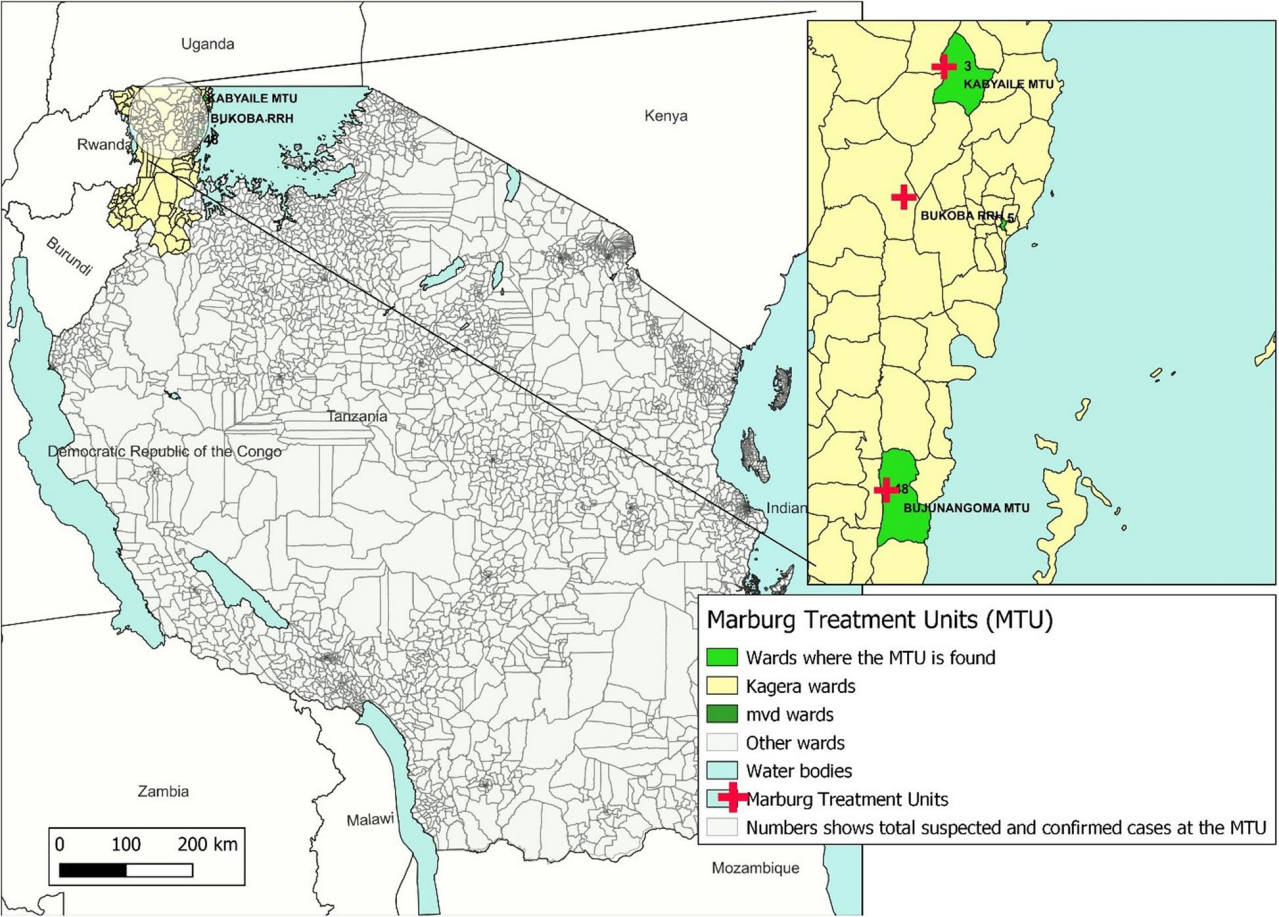
A map of Tanzania showing the MVD-affected areas and the established MTUs in Kagera region in 2023. Credit: Constructed by Atuganile, one of the co-authors using GIS software

Swift action was taken throughout the outbreak, with the identification of 212 contacts who may have been exposed to the disease. Because MVD has an incubation period that varies from 2 to 21 days [1], all contacts were monitored for 21 days. After 21 days of close monitoring [1], 53 individuals met the criteria as suspect cases, but only two exhibited transient MVD symptoms that eventually resolved. Regrettably, one individual passed away from unrelated causes. Notably, thanks to the prompt deployment of Rapid Response Teams, no patients succumbed to the disease after the declaration of MVD. As shown in Table 1; Fig. 1, live cases and suspects were managed in three designated facilities: Bukoba RRH (40), Kabyaile (3), and Bujunangoma (49). A suspect was defined as any person in Kagera with fever and bleeding from any orifice during the outbreak period. The national census of 2022 showed that the Kagera region had a population of 2,989,299, making it one of the 10 regions (out of 26 on the Tanzania mainland) with the highest population density; it has a population density of 118 persons per square kilometre.

**Table 1:** Number of Cases and Suspects Managed in each MTU: No suspect developed into a case, nor did any case result in death

Name of MTU	Number of confirmed cases^a^	Number of suspects	Suspects turned to cases
1. Bukoba RRH	1	3	0
2. Kabyaile	0	3	0
3. Bujunangoma	2	47	0
Total	3	53	0

^a^The number of cases doesn’t include the 6 deaths that occurred outside MTU before the declaration

#### The map of Tanzania showing the area of the affected Kagera Region

### How built preparedness in IPC was utilized to Control MVD in Kagera

After the MVD outbreak declaration, the Chief Government Medical Officer, also the default National Incident Manager, activated the Incident Management System (IMS) and deployed national and regional teams. These teams were acquainted, well-trained, and had guidelines directing their establishment and roles. The teams were structured into various pillars including case management and IPC [32]. More than 117 experts from various fields who had received training participated in the IPC and case management teams. The role of IPC in controlling the MVD outbreak has been summarized below using the four universal hierarchy of controls approach in preventing the transmission of infections within healthcare settings as stipulated by the United States Centers for Disease Control and Prevention (US CDC) [33].

#### First level of control: elimination of potential MVD exposures

The first and most effective hierarchy of control in MVD outbreak is the elimination of MVD exposure. In MVD context, the key strategy in elimination of exposures is limiting conduct of elective surgical procedures and regulation of general outpatient visits across all healthcare facilities. In the MVD outbreak of Kagera, patients typically originated from the community. Hence the Ministry conducted widespread public health campaigns and media outreach to enhance awareness of the circulating infection. Every client who presented at a healthcare facility was screened for cardinal signs and symptoms of MVD, such as fever. All suspected cases were isolated in special rooms where further monitoring took place. A campaign on safe and dignified burials for deceased MVD patients was conducted. The Ministry, in collaboration with local authorities and security agencies, ensured that national guidelines for safe and dignified burials were followed to limit MVD infections at the community level.

### Second level of control: administrative controls

In a battle against contagious diseases like MVD, the importance of administrative controls cannot be overstated. This second tier of infection control focuses on existing policies and procedures that aim to prevent the spread of the disease. By implementing these measures, healthcare facilities were able to protect their staff and patients. The key elements of administrative controls implemented during the control of this MVD outbreak included: early detection of cases, identifying IPC training and resource needs, training HCWs and distributing resources based on identified gaps, and ensuring supervised burial of every deceased regardless of the cause of the disease. All four of these measures are described below.

- **Early detection of cases**

To control MVD spread, early detection and ongoing surveillance of high-risk patients was required. Early outbreak detection is hindered by non-specific symptoms, leading to delays in diagnosis and increased transmission risk. Once an outbreak is identified, real-time infection confirmation is feasible but often necessitates a temporary laboratory near the outbreak area [34]. In this regard, the Ministry stationed a mobile laboratory in the Kabyaile Centre, located in the affected area. Operational definitions of suspected and probable cases were developed based on clinical and epidemiological factors.

To enhance the ability to detect cases, every patient who entered MTU was evaluated for probable infection. The contacts of each infected patient were tracked down in order to find more high-risk individuals who needed strict monitoring for sickness symptoms. All individuals who had recent direct physical touch with the patient, their bodily fluids, or their clothes or linens were included in this. By measuring the temperature at least once each day, all case contacts, including all medical staff who enter the isolation unit, were monitored for any signs of sickness. Regular temperature checks and extended monitoring periods allowed for the early identification of potential cases and enabled healthcare professionals to take appropriate actions in a timely manner. After the final known contact with the case, monitoring continued for an additional 21 days. Throughout the outbreak period, it was observed that none of the identified contacts displayed any symptoms. This indicated the effectiveness of the monitoring and preventive measures taken to contain the spread of the infection. By implementing these rigorous protocols, the healthcare professionals were able to effectively identify and monitor high-risk individuals, thereby minimizing the risk of further transmission.

b) **Identification of IPC training and resource needs**

The second administrative measure involved identifying IPC training and necessary resources for all HCWs before their assignment to MTUs. Initially, 241 HCWs from all healthcare facilities in the targeted areas (affected district and other two neighbouring districts) received comprehensive refresher training, which is equivalent to 43.3% of all 557 HCWs available in the Kagera region. This means that each facility in the targeted area had a staff member who would lead others in managing IPC activities. These staff members came from an area with a catchment population of 1,350,439, which is equivalent to 45.2% of the total population of Kagera.

Concurrently, a rapid assessment was conducted in the targeted area to determine the availability of IPC guidelines, Personal Protective Equipment (PPE), and other related commodities. A total of 29 healthcare facilities, encompassing both public and private facilities, were involved. This is equivalent to 21% of the total facilities available in the affected district council and the two neighbouring councils, amounting to 139 facilities in total. These included one regional referral hospital, six health centres, 14 dispensaries, and eight medical clinics. These facilities were selected because were at risk of receiving suspected clients. During this assessment, HCWs were mentored and provided with instructions and an understanding of IPC and patient care.

The guidance provided to HCWs during mentorship includes but is not limited to, the following IPC areas: hand hygiene, decontamination, waste management, traffic flow and activity patterns, use of PPEs, housekeeping, environmental cleaning and disinfection, linen and laundry, monitoring of HAIs, injection safety, safe and dignified burials, prevention of AMR, prevention and management of accidental exposure to blood borne pathogens. After the declaration of the end of the MVD, an After Action Review was conducted. This review led to the development and implementation of a plan to assess and mentor other health facilities at Kagera and other regions. Additionally, an isolation unit for highly infectious diseases that meets international standards was built in Kagera.

c) **Distribution of resources based on identified gaps**

Efficient resource allocation plays a pivotal role in ensuring the well-being of both patients and healthcare professionals. After needs assessment and capacity building, the third step involved distribution of SOPs, materials and supplies that are related to handwashing, chlorine mixing, waste management, laundry, cleaning and disinfection, PPE donning and doffing, safe burials, safe injections, MVD specimen collection criteria, analysis, diagnosis of suspects/patients and MVD discharge criteria.

d) **Discharge protocols and Burial Supervision regardless of the cause of the disease**

When it comes to the ongoing repercussions of a disease outbreak, there are crucial steps that need to be taken to ensure the safety of the community and the individuals affected. One such step is the development of discharge protocols for recovered patients or dead ones. In this area, the first task was to prepare a protocol for discharging recovered patients. In this protocol, for example, a person who has recovered from MVD would be allowed to go home after testing negative on a PCR test after 21 days. However, they would be advised to practice safe sex for the next 12 months because evidence shows that during this period, there is a risk of virus presence in semen. Clothes and materials used, for example, mobile phones, were incinerated and not allowed to leave the facility. Patients were provided with new clothes and materials, to replace destroyed items before going home. Everyone was required to shower before leaving the facility. Furthermore, HCWs were assured that they would receive excellent services in case they encountered any occupational hazards. All deaths in the affected regions were supervised with IPC experts who ensured dignified burials were conducted under safety measures.

#### Third level of control: engineering and environmental controls

The third level in the hierarchy focuses on Engineering and Environmental Controls. In the context of the MVD outbreak, which occurred in the wake of the COVID-19 pandemic, the Ministry, in collaboration with local authorities, took proactive measures. This included ensuring that every healthcare delivery point had fully equipped handwashing facilities. Tanzania has ten qualified MTUs for highly infectious diseases, and none of them are from the affected region. Nevertheless, three facilities within the region that fulfil the majority of MTU requirements were identified and subsequently upgraded to meet the basic standards for the treatment of MVD. Bujunangoma and Kabyaile were prepared for patients requiring uncomplicated care, while Bukoba RRH was designated for intensive care referrals. During the response, it was agreed that Kabyaile should no longer be used for patients but serve as a testing centre.

**Fig. 2:**
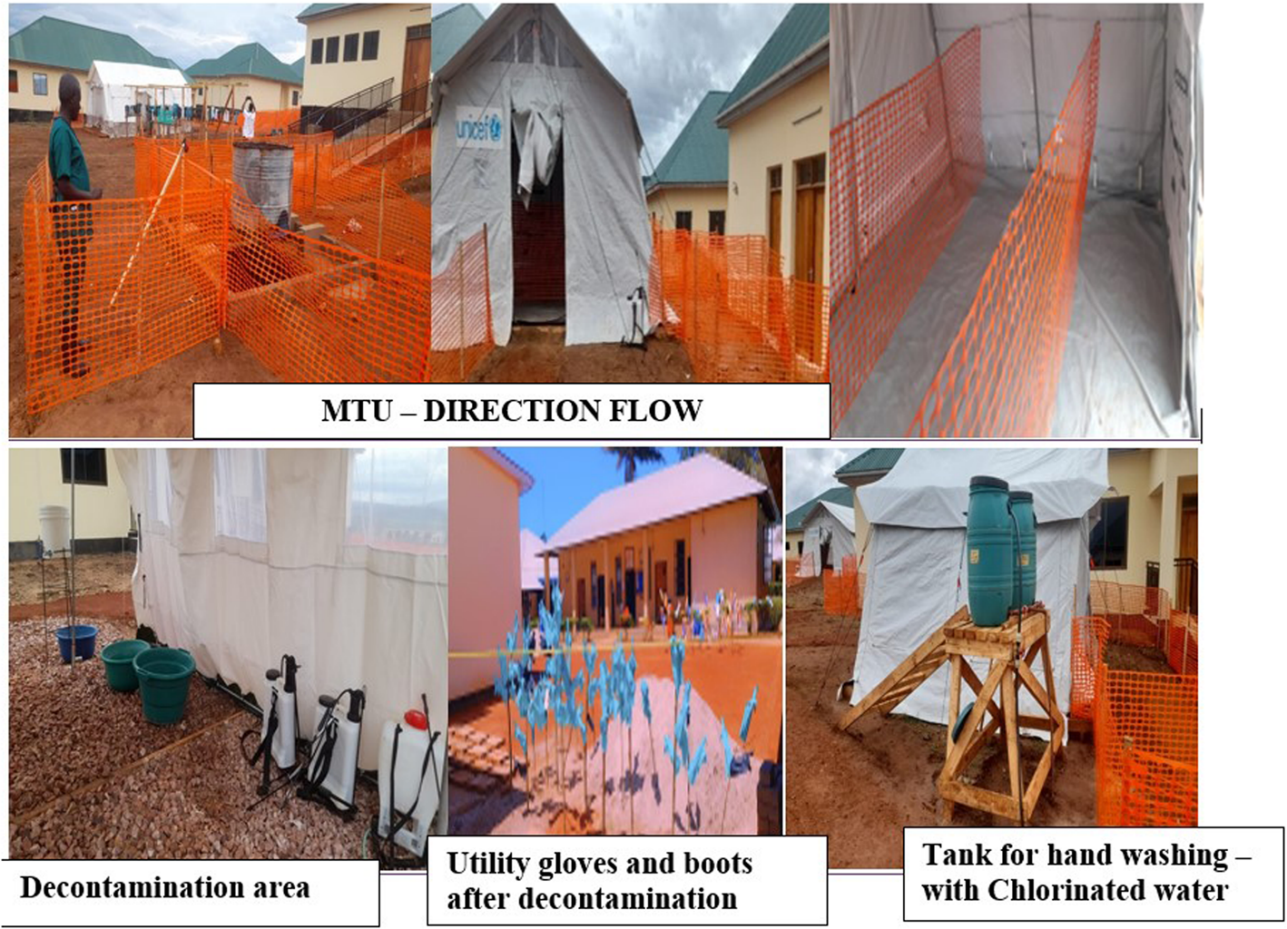
A picture of MTU showing some key features of importance for prevention and control of MVD at Kagera. Source: Taken by one of the co-authors

The layout of MTUs was required to comply with National IPC guidelines. Corrections were made whenever necessary. For instance, all MTUs had properly designated traffic flow to facilitate service provision and minimize transmission and were enclosed with a transparent fence to physically separate them from other units. Patient care wards were allocated space for funeral arrangements, outdoor areas for burning contaminated materials, clean storage for equipment, a resting area, and a pharmacy. The isolation wards were designed to provide ample space for separating confirmed cases from potential cases. Measures were taken to minimize crowding, reducing the risk of cross-contamination and creating a comfortable, secure workplace. To ensure a continuous supply of power and water for MTUs, standby electrical generators were installed (Fig. 2).

In every MTU there was an IPC expert who was providing guidance on how HCWs and the support staff should work based on IPC principles. His or her obligation was to make sure whoever has the task is paired with the buddy, decontamination is done based on SOPs, hand washing facilities are available, and all who are at MTU comply with hand hygiene and follow unidirectional principles.

Everything in the isolation unit, including human waste, was cleaned and disinfected before being removed. Effective disinfectants were used which included 0.05% chlorine solutions for disinfecting hands and linens; and 0.5% chlorine solutions for extremely contaminated objects like human excreta, body bags, major spills, and reusable PPE including boots, aprons, utility gloves, and googles. Items like mattresses that were difficult to sterilize were burnt. The isolation unit had easy access to sharps containers, and recapping needles was prohibited. After being removed from the isolation ward, contaminated non-disposable items were cleaned and disinfected. Additionally, the MTU’s surfaces underwent daily decontamination.

#### Fourth level of control: effective hand hygiene practices and effective use of PPE

The fourth level of implementation focused on promoting excellent hand hygiene practices and the effective utilization of PPE. Performing invasive procedures to save the life of a pregnant woman with VHF infection was not recommended [10] for various reasons, including the difficulty in controlling infections for HCWs. However, with robust training to the team and heightened motivation, the team successfully saved the life of a pregnant woman with MVD infection experiencing an incomplete abortion within MTU. This success was attributed to the team’s confidence, facilitated by the availability of all necessary MVD-protective PPE in the facility. The positive outcome of the MVA procedure was ensured through strict IPC observations. The budding system was mandatory for everyone who donned PPE and did any procedure like clinical care, nursing care, cleaning, etc. The system involved pairing of the teams whereby one person was solely watching the other person who was performing the procedure so that he or she was adhering to all the IPC principles.

Significant emphasis was placed on ensuring effective hand hygiene at all five moments requiring hand hygiene in MTUs. The five moments for hand hygiene are: before touching a patient, before a procedure, after a procedure or exposure to body fluids, after touching a patient, and after touching a patient’s surroundings. Effectiveness entails handwashing with soap and water or the use of alcohol-based sanitizers, following established national and international SOPs.

## Discussion

This section discusses how IPC practices prevented further transmission in Marburg Virus survivors. It also explores the possible contributing factors that protected relatives who cared for the initial deceased cases with limited adherence to IPC measures, and, finally, we discuss limitations in adherence to IPC during the MVD outbreak in Kagera.

In this MVD outbreak, healthcare workers implemented and maintained stringent IPC practices, which effectively minimized the risk of transmission among long-term survivors. Various strategies were implemented. Implementing strict hand hygiene measures, including frequent handwashing with soap and water or the use of alcohol-based sanitisers was the primary strategy. This simple, yet effective, measure significantly reduced the risk of transmitting the virus from contaminated surfaces to individuals. Proper use of personal protective equipment (PPE), including wearing gloves, gowns, masks, and eye protection helped minimize the risk of direct contact with bodily fluids that may have contained the virus. Additionally, isolating infected individuals in designated areas further prevented transmission to other patients or healthcare workers.

When the initial cases of MVD surfaced, some relatives stepped up to care for their infected family members. Some of these caregivers did not adhere to IPC measures but remained unaffected. We believe that genetic predisposition and genetic variations among individuals might have played a role in granting enhanced immunity against the Marburg Virus. Previous studies have documented that host factors such as genetic susceptibility, past exposure to cross-reactive agents and the presence of concurrent infections, and the age of the host when infected can influence the pattern and outcome of viral infections, including MVD [35, 36]. It is possible that these immune responses were particularly robust in the relatives who escaped infection, effectively neutralizing the virus before it acted. Furthermore, environmental factors could have also played a role. Neutralizing antibody responses have been described in cases of a single case of MVD infection [37, 38].

The implementation of IPC interventions to manage MVD in Kagera faced specific challenges. A significant hurdle was encountered when HCWs attended to MVD patients requiring mental healthcare while donning full PPE. Although it is advisable for providers not to wear full PPE for more than an hour within the MTU to mitigate occupational thermal risks, this particular category of clients necessitates extended care. The approach taken was to communicate to the patients the difficulty of providing prolonged care with a single provider. Consequently, when necessary, two providers were utilized, taking turns to ensure continuous and appropriate care.

Another challenge arose initially as some providers expressed reluctance to be deployed to treatment units due to concerns about contracting infections. However, the majority’s fear diminished after receiving sufficient counselling and assurances of the implementation of policies to safeguard their safety. This included assurance of unlimited and better services in the event of provider infection, improving the working environment, and ensuring the availability of the required PPE.

### Conclusion and recommendations

The MVD outbreak in Kagera has served as a valuable lesson, demonstrating that the spread of highly infectious diseases can be effectively controlled through effective preparedness and rigorous adherence to IPC practices. While it cannot be definitively asserted that IPC measures alone facilitated the control of this disease [1], its contribution included instilling confidence among HCWs who voluntarily undertook high-risk procedures, such as MVA, to preserve the lives of infected mothers experiencing incomplete abortion. A further strategy to disrupt the transmission cycle of MVD involved restricting all burial services under the oversight of IPC experts.

This report provides an example of how other low-income countries can learn to prepare for and address highly infectious disease outbreaks through the effective implementation of IPC measures. This report also adds to the literature on the lessons and initial response actions to the MVD outbreak in Tanzania in 2023 [31, 39–42].

Kagera is a region bordering three East African countries: Uganda, Rwanda, and Burundi. Without effective measures to control this disease, there was a potential risk of its spread to neighbouring countries and other regions of Tanzania. In line with the implementation of IPC practices, these countries are advised to seek various means to motivate frontline HCWs including incentives, improved working conditions, health insurance, and training, to encourage their full commitment to serving clients in challenging environments.

## Data Availability

All data generated or analysed during this study are included in this published article.
